# Effects of Worsening Renal Function and Changes in Blood Urea Nitrogen Level During Hospitalization on Clinical Outcome in Patients with Acute Decompensated Heart Failure

**DOI:** 10.3390/biomedicines13040977

**Published:** 2025-04-16

**Authors:** Masaru Hiki, Takatoshi Kasai, Akihiro Sato, Sayaki Ishiwata, Shoichiro Yatsu, Hiroki Matsumoto, Jun Shitara, Megumi Shimizu, Azusa Murata, Takao Kato, Shoko Suda, Hiroshi Iwata, Atsutoshi Takagi, Hiroyuki Daida

**Affiliations:** 1 Department of Cardiovascular Medicine, Juntendo University Graduate School of Medicine, Tokyo 113-8421, Japan; ma-hiki@juntendo.ac.jp (M.H.); ak-sato@juntendo.ac.jp (A.S.); s-ishiwata@juntendo.ac.jp (S.I.); syatsu@juntendo.ac.jp (S.Y.); hmatsumo@juntendo.ac.jp (H.M.); jshitara@juntendo.ac.jp (J.S.); shimizumegumi1986@gmail.com (M.S.); azmurata@juntendo.ac.jp (A.M.); tkatou@juntendo.ac.jp (T.K.); ssuda@juntendo.ac.jp (S.S.); h-iwata@juntendo.ac.jp (H.I.); a.taka@juntendo.ac.jp (A.T.); daida@juntendo.ac.jp (H.D.); 2 Cardiovascular Respiratory Sleep Medicine, Juntendo University Graduate School of Medicine, Tokyo 113-8421, Japan; 3 Sleep and Sleep-Disordered Breathing Center, Juntendo University Hospital, Tokyo 113-8421, Japan; 4 Department of Cardiovascular Management and Remote Monitoring, Juntendo University Graduate School of Medicine, Tokyo 113-8421, Japan

**Keywords:** worsening renal function, blood urea nitrogen, acute decompensated heart failure, creatinine, mortality, rehospitalization

## Abstract

**Background/Objectives:** Worsening renal function (WRF) during hospitalization for acute decompensated heart failure (ADHF) is associated with poor clinical outcomes. Data on the impact of WRF on clinical outcomes, considering blood urea nitrogen (BUN) level and its changes in patients with ADHF, are scarce. This study aimed to investigate the effects of BUN and its changes during hospitalization on the relationship between WRF during hospitalization and post-discharge clinical outcomes in patients with ADHF. **Methods:** A total of 509 patients with ADHF, hospitalized between 2007 and 2011, were included. WRF was defined as an absolute increase in serum creatinine level of >0.3 mg/dL, with a >25% increase during hospitalization. The risk of WRF for post-discharge clinical events, including death and rehospitalization, considering BUN levels, was assessed using three multivariable Cox regression models. **Results:** WRF was observed in 55 (10.8%) patients. The cumulative event-free survival was significantly worse in patients with WRF (*p* = 0.039). In Model 1 (excluding BUN changes), WRF was associated with a greater risk of post-discharge clinical events. In Model 2, which included both WRF and BUN changes, WRF was not a significant predictor. In Model 3, patients were subdivided according to WRF or BUN increase, and the subgroups were included instead of isolated WRF and BUN changes; only WRF with increased BUN level was associated with an increased risk of post-discharge clinical events. **Conclusions:** In patients with ADHF, WRF was associated with poor post-discharge clinical outcomes when accompanied by increased BUN levels during hospitalization.

## 1. Introduction

In the long-term clinical course of heart failure (HF), managing acute worsening heart failure (acute decompensated HF [ADHF]) is important [1] because such episodes lead to further HF progression [2] and have become a major burden on healthcare systems worldwide. Thus, it is important to identify factors that reveal obvious changes in the acute phase of HF, which are associated with poor clinical outcomes [3]. In-hospital worsening of renal function (WRF) can be one such factor.

HF is closely associated with renal impairment in the acute and chronic phases [4]. Multiple mechanisms may explain such associations, including changes in glomerular hemodynamics in association with arteriolar hypoperfusion due to hyperactivity of the renin–angiotensin system (RAS) and sympathetic nerve system, renal venous congestion, drug-related adverse effects, and permanent nephron damages [5]. From this viewpoint, chronic kidney disease (CKD) is a well-known risk factor for poor clinical outcomes in patients with ADHF [6]. However, regardless of the presence or absence of CKD, an increase in serum creatinine level compared with the admission level is also common during hospitalization for ADHF [4]. This is referred to as WRF based on the increase in serum creatinine levels [6,7]. Although many studies have shown relationships between WRF and poor clinical outcomes in patients with ADHF, the impact on outcomes of WRF during hospitalization for ADHF remains controversial [8,9].

Blood urea nitrogen (BUN) is another available biomarker of renal function, which is a routinely measured, cost-effective biomarker of renal function and an indicator of clinical outcomes. The metabolism of creatinine and BUN are similar in that both are freely filtered at the glomerulus, but only BUN, not creatinine, is reabsorbed by approximately 50% in the tubules [10,11]. Since neurohumoral activation due to ADHF, including activation of RAS, sympathetic nerve system, and arginine vasopressin (AVP), directly or indirectly increases this reabsorption process independent of glomerular filtration [11], BUN and its changes during hospitalization are regarded as a prognostic indicator of poor clinical outcome in patients with ADHF independent of the creatinine level and its changes [10,12]. However, data regarding the impact of WRF on clinical outcomes, considering BUN and its changes, in patients with ADHF are scarce.

Therefore, this study aimed to investigate the effects of BUN and its changes during hospitalization on the relationship between WRF during hospitalization and post-discharge clinical outcomes in patients with ADHF.

## 2. Materials and Methods

### 2.1. Patients

This observational study utilized a prospectively collected database, targeting consecutive patients hospitalized in the cardiac intensive care unit at Juntendo University Hospital, Tokyo, Japan, between 2007 and 2011, who had a diagnosis of ADHF. ADHF was defined based on the modified Framingham criteria [13]. Patients who had acute coronary syndrome and/or had undergone cardiac surgery during the previous 4 weeks or during initial hospitalization, end-stage renal disease requiring dialysis, or a life-threatening malignancy were excluded. In addition, patients who died during their initial hospitalization and those without documented creatinine or BUN levels at admission and/or discharge were excluded. The Institutional Review Board of Juntendo University Hospital approved the study protocol (#871), and this study complied with the Declaration of Helsinki. Informed consent was obtained from all the patients.

### 2.2. Data Collection

Baseline data were prospectively collected during the initial hospitalization period. Medical history was obtained from patients’ clinical chart reviews. Body mass index (BMI) was calculated based on height and weight. Renal function was defined as the estimated glomerular filtration rate (eGFR), which was calculated using the modification of diet in renal disease equation with the Japanese coefficient from baseline serum creatinine levels [14]. Two-dimensional echocardiography was performed on each patient. Left ventricular ejection fraction (LVEF) was calculated using the modified Simpson method. WRF was defined as an absolute increase in serum creatinine level > 0.3 mg/dL in combination with >25% increase during hospitalization [7]. Data were collected on admission and at discharge. All patients were followed up from the date of index admission until December 2012. Outcome data were obtained by reviewing the medical records of our hospital for all deaths recorded after discharge and rehospitalization due to ADHF. We set the primary endpoint of the present study as a composite of all-cause mortality and rehospitalization due to ADHF (clinical event).

### 2.3. Statistical Analysis

Continuous variables are expressed as the mean ± standard deviation or median and interquartile range. Categorical variables are presented as numbers and percentages. Comparing the characteristics between patients with and without WRF, the χ^2^ test was used for categorical variables, the t-test for normally distributed continuous variables, and the Mann–Whitney U test for non-normally distributed continuous variables. Differences between admission and discharge measurements, such as BMI, LVEF, and BUN, serum creatinine, and plasma B-type natriuretic peptide (BNP) levels, were compared using the paired t-test for normally distributed data and the Wilcoxon signed-rank test for non-normally distributed data. Changes in parameters from admission to discharge (i.e., Δ) were computed as Δ = [(values at discharge) − (values at admission)] and expressed as %Δ [Δ/(values at admission) × 100]. The relationship between %Δ in BUN and %Δ in creatinine was assessed using the Spearman correlation coefficient. Cumulative survival curves following discharge in patients with and without WRF are depicted using the Kaplan–Meier method and compared using the log-rank test. Univariable Cox proportional hazards regression analysis was used to identify the association between clinical events and variables obtained at discharge, including age, sex, BMI, %Δ in BMI, history of HF, ischemic etiology, atrial fibrillation, diabetes mellitus (DM), systolic and diastolic blood pressure, heart rate, LVEF, %Δ in LVEF, hemoglobin level, eGFR, BUN level, %Δ in BUN, serum sodium and potassium levels, plasma BNP level, %Δ in BNP, use of HF medications, initiation of diuretics during hospitalization, and transient renal replacement therapy in addition to the presence or absence of WRF. Variables with *p* values < 0.1 in each univariable analysis were then included in a multivariable Cox proportional hazards regression analysis, except the %Δ in BUN, to confirm whether WRF can be a risk factor for clinical events (Model 1). Because we focused on the effects of BUN changes during hospitalization on the relationship between WRF and post-discharge clinical outcomes, %Δ in BUN was then entered into the abovementioned multivariable Cox proportional hazards regression analysis (Model 2). First-order interactions in the multivariable Cox proportional hazards models were examined by entering the interaction terms between the WRF and BUN changes. In addition, patients in each group with and without WRF were divided into two subgroups based on the presence or absence of BUN increases (%Δ > 0% or ≤0%), resulting in four subgroups: WRF-/BUN increase-, WRF-/BUN increase+, WRF+/BUN increase-, and WRF+/BUN increase+. Tertiary multivariable analysis included these subgroups instead of WRF and %Δ in BUN (Model 3). Because of the non-normal distribution, BNP levels were naturally log-transformed. The proportional hazards assumption was assessed using a log-minus-log survival graph. Differences were considered statistically significant at *p* < 0.05. All statistical analyses were performed using a statistical software package (SPSS version 23 SPSS Inc., Armonk, NY, USA).

## 3. Results

### 3.1. Patient Characteristics

A total of 751 patients with ADHF were admitted to our institution between 2007 and 2011. Among them, 190 patients with concomitant acute coronary syndrome and/or those who had undergone cardiac surgery during the previous 4 weeks, end-stage renal disease requiring dialysis, or life-threatening malignancy were initially excluded. Forty-seven patients who died during the initial hospitalization and five patients without serum creatinine and/or BUN values on admission and/or at discharge were also excluded. Thus, the data of 509 patients were analyzed. The patients were classified into two groups according to the presence or absence of WRF.

Patient characteristics at discharge are shown in Table 1. Patients with WRF (N = 55, 10.8%) had more impaired renal function, as indicated by higher BUN and creatinine levels and lower eGFR at discharge, compared to those without WRF. Patients with WRF were less likely to take angiotensin-converting enzyme inhibitors or angiotensin II receptor blockers, which was possibly associated with slightly higher serum potassium levels and more impaired renal function, although they had higher BNP levels than those without WRF. Diuretics were initiated during hospitalization in 21 patients with WRF (38.2%) and 185 patients without WRF (40.7%) (*p* = 0.825). Transient renal replacement therapy was performed during hospitalization in 10 patients with WRF (18.2%) and 46 patients without WRF (10.1%) (*p* = 0.116).

### 3.2. Changes in BMI, LVEF, BUN, Creatinine, and BNP Levels

Changes in parameters from admission to discharge and %Δ are summarized in Table 2. In patients without WRF, the BUN, creatinine, and BNP levels decreased significantly from admission to discharge. However, despite the significant decrease in BNP levels in patients with WRF, significant increases in BUN and creatinine levels were observed from admission to discharge. Indeed, %Δ in BUN and creatinine indicated significantly greater increases in both of them compared with those without WRF. However, there was only a weak correlation between %Δ in BUN and %Δ in creatinine (correlation coefficient, 0.114; *p* = 0.010).

### 3.3. Outcomes

**Figure 1:**
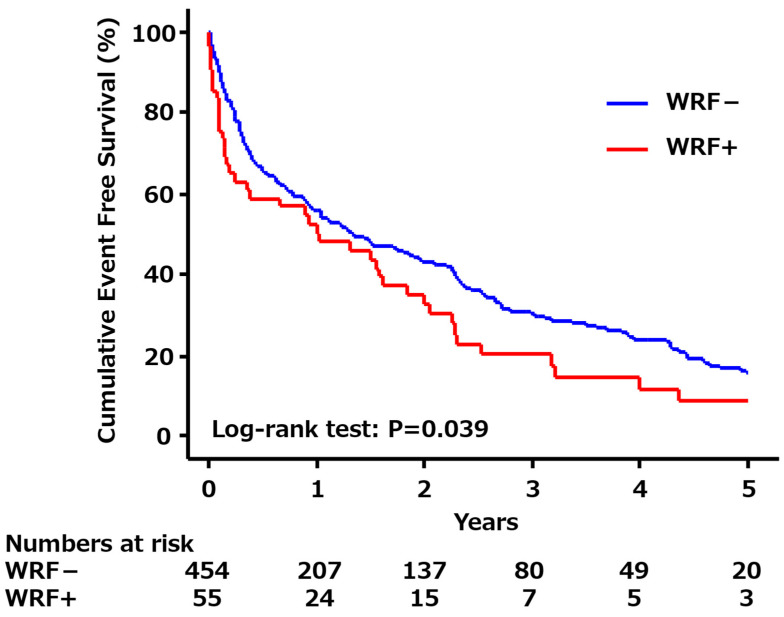
Cumulative event-free survival curves according to the presence or absence of WRF.

The median follow-up period was 1.4 years. During follow-up, 331 clinical events (65.0%), including 131 deaths (25.7%) and 200 rehospitalizations (39.3%), were observed in 509 patients: 289 clinical events (63.7%), including 111 deaths (24.4%) and 178 rehospitalizations (39.2%) in patients without WRF, and 42 clinical events (76.4%), including 20 deaths (36.4%) and 22 rehospitalizations (40.0%) in patients with WRF. There was a significant difference in the cumulative survival curves between the patients with and without WRF (log-rank test; *p* = 0.039) (Figure 1). Univariable Cox proportional hazard analyses revealed that age, female sex, ischemic etiology, DM, use of beta blockers, initiation of diuretics during hospitalization, transient renal replacement therapy, %Δ in BUN, and WRF are correlated with an increased risk of post-discharge clinical events (*p* < 0.1). In multivariable analysis, including those variables except for %Δ in BUN (Model 1), the presence of WRF was significantly associated with an increased risk of post-discharge clinical events along with an increase in age, presence of DM, initiation of diuretics during hospitalization, and transient renal replacement therapy (Table 3). However, when %Δ in BUN was added to the multivariable model (Model 2), only an increase in age, presence of DM, initiation of diuretics during hospitalization, and transient renal replacement therapy were significantly associated with an increased risk of post-discharge clinical events, and presence of WRF was no longer a significant factor (Table 3). No significant interaction was noted between %Δ in BUN and WRF (*p* for interaction, 0.339). Results of tertiary multivariable analysis, in which four subgroups instead of %Δ in BUN and WRF were included (Model 3), indicated that only patients with BUN increase and WRF during hospitalization had the risk of post-discharge clinical events in addition to greater age, presence of DM, initiation of diuretics during hospitalization, and transient renal replacement therapy (Table 3).

In patients with WRF, the cumulative event-free survival is significantly worse than in those without WRF (log-rank test: *p* = 0.039). WRF, worsening renal function.

## 4. Discussion

The findings of this study offer important insights into the association between WRF and post-discharge clinical outcomes in patients with ADHF. First, patients with WRF had an increased risk of death or rehospitalization due to ADHF, even in multivariable analysis, which is consistent with previous studies. Second, BUN changes from admission to discharge, but not the BUN level at discharge, were associated with an increased risk of post-discharge clinical events in the univariable analysis. When BUN changes were included in the multivariable analysis, the risk of either WRF or BUN changes was no longer significant. Finally, when patients were categorized into four groups based on the presence or absence of WRF and BUN increase, and when such a four-group category was included in the multivariable analysis instead of isolated WRF and BUN changes, only the combined BUN increase and WRF were associated with an increased risk of post-discharge clinical events. These findings suggest that WRF can predict an increased risk of post-discharge mortality and rehospitalization in patients with ADHF; however, an increase in BUN levels may play a significant role in the relationship between WRF and post-discharge clinical outcomes.

Impaired renal function is a common comorbidity in patients with both ADHF and chronic HF, regardless of reduced or preserved LVEF [3,4], possibly through the multiple mechanisms such as changes in glomerular hemodynamics in association with activated RAS and sympathetic nerve system, congestions in renal veins in association with systemic congestion, adverse effects of some medications for HF, and organic damage in the kidneys [5]. Although CKD is a common comorbidity and one of the prognostic factors even in patients with ADHF [6], WRF, an increase in serum creatinine level from the admission level, is observed during hospitalization for ADHF in approximately 10–20% of patients with ADHF [4,6,7]. Consistent with previous studies, our study suggested that WRF can be a predictor of poor post-discharge clinical outcomes in patients with ADHF [8]. However, whether WRF during hospitalization for ADHF itself increases the risk for poor post-discharge clinical outcomes remains controversial because WRF can be a reflection of congestion, adverse effects of HF medications, or hemodynamic instability [8,9,15]. Thus, other indicators of renal function, such as BUN, are in focus.

Creatinine and BUN are freely filtered at the glomerulus, but only BUN, not creatinine, is reabsorbed by approximately 50% in the tubules [10,11]. ADHF activates the systemic RAS and sympathetic nervous system, and in the kidney, the reabsorption of sodium and water increases in the proximal tubule, leading to increased urea concentration in the proximal tubule and decreased urine flow in the collecting duct, both leading to an increase in urea reabsorption [11,16]. In addition, arterial underfilling associated with ADHF increases baroreceptor-mediated AVP release, which upregulates urea transporters in the inner medullary collecting duct. Thus, neurohumoral activation may increase BUN levels independent of a decrease in glomerular filtration rate, which is usually expressed as an increase in serum creatinine levels. Considering this, BUN may reflect neurohormonal activation [16,17], which can also worsen clinical outcomes in patients with ADHF [18]. Indeed, BUN has a prognostic impact in patients with ADHF independent of creatinine level [10]. In addition, an increase in BUN during hospitalization has a prognostic impact on patients with ADHF [12]. However, the impact of WRF on clinical outcomes, considering BUN and its changes, in patients with ADHF has been rarely investigated. Palazzuoli et al. reported that an increase in BUN of ≥20% during hospitalization for ADHF predicts a poor outcome independent of WRF and that WRF predicts adverse outcomes only if BUN increases substantially [19]. Our findings are in line with theirs; however, the differences between ours and theirs are that the percentage increase in BUN during hospitalization for ADHF was higher and that WRF was defined as an in-hospital increase in serum creatinine level of ≥0.3 mg/dL or an eGFR reduction of ≥20% in their study. Nevertheless, since BUN changes in WRF have not yet been considered in recent clinical settings, we believe the findings of the present study are worth reporting despite using an outdated dataset.

This is the first study to show that WRF predicts adverse post-discharge outcomes only when accompanied by the neurohumoral activation indicated by an increased BUN level. Considering this, beyond the current guideline-directed medical therapies for ADHF with WRF [20], the addition of neurohumoral blockades such as AVP blockade using tolvaptan or the supplementation of natriuretic peptide with loop diuretics may have some impacts in terms of improving the post-discharge clinical outcome in the subset of patients with ADHF who have WRF and increased BUN levels during hospitalization, although their benefits with respect to post-discharge clinical outcomes were not established, overall, in patients with ADHF [21,22]. Further studies investigating whether the selective use of such therapy in the subset of patients with ADHF is effective in reducing the risk of post-discharge clinical events are needed.

Our study has some limitations. First, it was conducted at a single academic center and involved a limited number of patients and clinical events. Second, because the present study was observational, other confounders that might have affected the results (e.g., the presence of osteoarthritis and its treatments, tumor disease, except for life-threatening malignancy, and gastrointestinal bleeding, cannot be ruled out, even after the adjusted analysis). Moreover, recent advances in HF and renal protective therapies, such as sacubitril–valsartan, sodium–glucose cotransporter 2 inhibitors, and new mineralocorticoid receptor antagonists, may affect the results of the present study [23]. Third, other indicators of renal function, such as cystatin C levels, were not considered. Changes in these indicators may also affect the association between WRF and clinical outcomes [4]. Fourth, although the significant point of %Δ in BUN in identifying poor post-discharge clinical outcomes is of interest, the numbers of patients and clinical events in patients with WRF were too small to assess this point of %Δ in BUN. Further studies on a large number of patients with WRF are required. Fifth, although diastolic dysfunction plays some role in the relationship between WRF, increased BUN levels, and clinical outcomes [24], data on diastolic function was not assessed in this study. Finally, although we considered medication use at discharge in the multivariable analysis, changes in medications and their dosages were not considered.

## 5. Conclusions

We found that WRF can be a predictor of an increased risk of mortality and rehospitalization in patients with ADHF; however, an increase in BUN during hospitalization may play an important role in the relationship between WRF and post-discharge clinical outcomes. These findings may increase awareness of the clinical importance of monitoring changes in BUN levels during hospitalization for ADHF.

## Figures and Tables

**Table 1:** Patient characteristics at discharge.

	Without WRF N = 454	with WRF N = 55	*p*
Age, years	70.4 ± 14.0	70.9 ± 12.4	0.798
Women, *n* (%)	166 (36.6)	19 (34.5)	0.884
BMI, kg/m^2^	22.1 ± 6.6	21.9 ± 7.4	0.864
History of HF, *n* (%)	230 (50.7)	29 (52.7)	0.883
Ischemic etiology, *n* (%)	182 (40.1)	21 (38.2)	0.899
AF, *n* (%)	177 (39.0)	18 (32.7)	0.450
Diabetes mellitus, *n* (%)	164 (36.1)	26 (47.3)	0.142
Systolic BP, mmHg	114.8 ± 17.5	111.0 ± 17.3	0.148
Diastolic BP, mmHg	62.1 ± 9.9	59.9 ± 11.4	0.146
HR, beats/min	71.7± 10.8	74.1 ± 14.3	0.255
LVEF, %	43.9 ± 17.2	39.6 ± 18.0	0.231
LVEF ≤ 40%, *n* (%)	220 (48.5)	30 (54.5)	0.437
Hemoglobin, g/dL	11.9 ± 2.3	11.3 ± 2.6	0.339
BUN, mg/dL	24.8 ± 14.6	43.5 ± 30.0	0.048
Creatinine, mg/dL	0.9 [0.5]	1.5 [1.3]	<0.001
eGFR, mL/min/1.73 m^2^	53.8 [33.2]	31.7 [24.8]	<0.001
Sodium, mmol/L	138.6 ± 4.0	139.3 ± 4.9	0.243
Potassium, mmol/L	4.3 ± 0.5	4.5 ± 0.6	0.007
BNP, pg/mL	259.4 [374.0]	406.2 [658.0]	0.007
Beta blockers, *n* (%)	295 (65.0)	29 (52.7)	0.102
ACE-Is/ARBs, *n* (%)	299 (65.9)	28 (50.9)	0.042
Aldosterone blockers, *n* (%)	161 (35.5)	16 (29.1)	0.431
Diuretics, *n* (%)	319 (70.3)	34 (61.8)	0.259

Variables are expressed as the mean ± standard deviation, median [interquartile range] or *n* (%). AF, atrial fibrillation; ACE-I, angiotensin-converting enzyme inhibitor; ARB, angiotensin II receptor blocker; BMI, body mass index; BNP, B-type natriuretic peptide; BUN, blood urea nitrogen; BP, blood pressure; eGFR, estimated glomerular filtration rate; HF, heart failure; HR, heart rate; LVEF, left ventricular ejection fraction; WRF, worsening renal function.

**Table 2:** Changes in the values between admission and discharge.

	WRF	Admission	Discharge	*p*	%Δ	*p* for %Δ
BMI, kg/m^2^	No	23.0 ± 5.1	22.1 ± 6.6	0.075	−8.0 [43.0]	0.617
	Yes	22.0 ± 4.0	21.9 ± 7.4	0.936	1.8 [45.3]	
LVEF, %	No	42.7 ± 17.5	43.9 ± 17.2	0.293	2.2 [90.8]	0.069
	Yes	44.9 ± 18.3	39.6 ± 18.0	0.139	−17.0 [73.7]	
BUN, mg/dL	No	27.1 ± 16.9	24.8 ± 14.6	0.001	−5.3 [46.9]	<0.001
	Yes	21.8 ± 11.1	43.5 ± 30.0	<0.001	76.5 [120.8]	
Creatinine, mg/dL	No	1.0 [0.7]	0.9 [0.5]	<0.001	−8.4 [85.5]	<0.001
	Yes	1.1 [1.3]	1.5 [1.3]	0.026	38.8 [149.3]	
BNP, pg/mL	No	641.6 [905.6]	265.4 [386.0]	<0.001	−61.2 [88.6]	0.129
	Yes	534.4 [933.6]	383.6 [580.0]	0.021	−41.5 [105.7]	

Variables are expressed as the mean ± standard deviation or median [interquartile range]. BMI, body mass index; BNP, brain natriuretic peptide; BUN, blood urea nitrogen; LVEF, left ventricular ejection fraction; WRF, worsening of renal function.

**Table 3:** Results of univariable and multivariable Cox proportional hazards regression analyses.

	Univariable			Multivariable Model 1			Multivariable Model 2			Multivariable Model 3		
	HR	95% CI	*p*	HR	95% CI	*p*	HR	95% CI	*p*	HR	95% CI	*p*
Age - 1 y inc	1.02	1.01–1.03	<0.001	1.02	1.01–1.03	0.002	1.02	1.01–1.02	0.003	1.02	1.01–1.03	0.002
Women - yes	1.22	0.98–1.53	0.077	1.22	0.97–1.54	0.092	1.20	0.95–1.52	0.133	1.22	0.97–1.55	0.092
Ischemic etiology - yes	1.35	1.09–1.68	0.006	1.08	0.85–1.36	0.550	1.07	0.85–1.36	0.563	1.08	0.85–1.37	0.545
DM - yes	1.65	1.32–2.06	<0.001	1.56	1.23–1.98	<0.001	1.56	1.23–1.98	<0.001	1.57	1.24–1.99	<0.001
Beta-blockers - yes	1.23	0.98–1.55	0.073	1.24	0.98–1.56	0.078	1.12	0.98–1.57	0.071	1.23	0.97–1.55	0.088
Initiation of diuretics during hospitalization - yes	0.76	0.61–0.95	0.015	0.78	0.62–0.98	0.030	0.78	0.62–0.98	0.033	0.77	0.62–0.97	0.026
Transient renal replacement therapy - yes	1.68	1.22–2.31	0.001	1.55	1.12–2.15	0.008	1.55	1.12–2.15	0.008	1.55	1.12–2.14	0.009
%Δ in BUN −1% inc	1.01	1.00–1.01	0.010	-	-	-	1.00	0.99–1.00	0.240	-	-	-
WRF - yes	1.40	1.02–1.94	0.040	1.42	1.03–1.97	0.035	1.28	0.87–1.87	0.209	-	-	-
Four-group												
WRF- BUN inc−	Reference			-	-	-	-	-	-	Reference		
WRF- BUN inc+	1.12	0.89–1.42	0.341	-	-	-	-	-	-	1.12	0.88–1.42	0.357
WRF+ BUN inc−	1.23	0.46–3.33	0.677	-	-	-	-	-	-	1.48	0.55–4.02	0.438
WRF+ BUN inc+	1.51	1.06–2.14	0.023	-	-	-	-	-	-	1.49	1.05–2.13	0.028

BUN, blood urea nitrogen; CI, confidence interval; DM, diabetes mellitus; HR, hazard ratio; WRF, worsening of renal function; inc, increase.

## Data Availability

The data supporting this study’s findings are available from the corresponding author upon reasonable request.

## Abbreviations

The following abbreviations are used in this manuscript:

WRF	Worsening renal function
ADHF	Acute decompensated heart failure
HF	Heart failure
CKD	Chronic kidney disease
RAS	Renin–angiotensin system
AVP	Arginine vasopressin
BMI	Body mass index
eGFR	Estimated glomerular filtration rate
LVEF	Left ventricular ejection fraction
BNP	B-type natriuretic peptide
DM	Diabetes mellitus
